# The Growth of Hospital Opinion

**Published:** 1888-06-16

**Authors:** 


### The Hospital

A Weekly Institutional Journal of

Science, Medicine, Nursing, and Philanthropy.

*For Hospitals, Asylums, Medical Practitioners, Students, Nurses, Families, Parishes, Congregations, and the Charitable Public.*

Vol. IV. No. 90] [June 16, 1888.

### The Growth of Hospital Opinion.

We are reminded as we write that neither the food supplied nor the washing allowed to nurses in hospitals twenty years ago was much thought of. Indeed, the term "probationer nurse" was not then in use, and as for training, the nurses of those days were for the most part, in this respect, like Topsy in "Uncle Tom's Cabin"—they "growed" into nurses as best they could. As with nurses so with hospital affairs in general, for these the great outside public had very little concern or thought. Managed for the most part by the few who absorbed the patronage, such as it was, many hospitals became close boroughs of a very exclusive and narrow kind. Hospital Sunday was hardly known or heard of outside its birthplace, Birmingham; whilst Hospital Saturday was not even instituted. Twenty years has changed many things, but few public institutions have undergone so great a change in the public estimation as hospitals and nursing institutions. Then they were recognised indeed as isolated and necessary aids to the life of every community of importance, but as for their being part of a great system of relief to which all owed much, and by which all must benefit, this was not realized, if it was even dreamt of by the public. Now, as the present week has shown, statesmen of the first rank, peers, politicians, and professional men of all grades have come to see that our hospital system must be maintained, and that the voluntary method is far preferrable to any other that can be conceived.

What has brought about this change in the public sentiment towards our hospitals? The Hospital Sunday Fund organization has done something to promote it, and the editors of the leading journals have nobly seconded the efforts of the clergy and ministers to arouse and fix the people's attention upon the needs and work of hospitals. Last week the *Times* newspaper, with a generosity worthy of all praise, devoted several columns of space to the cause, and that, day after day, throughout the Hospitals Week. Its efforts, were well supported by the *Morning Post*, the *Daily Telegraph*, the *Daily News*, and the *Daily Chronicle*, the editors of which gave a good share of space on more than one day during this week of philanthropic effort whilst the *Star* and the *Echo* especially, and the *Pall Mall* and *St. James's Gazettes*, to a less extent, co-operated in the movement with generous cordiality. Of course there were one or two editors who found the pressure on their space so great as to prevent them from joining the movement this year, but we hope all will lend a hand on the next occasion. It remains, to be seen how far this united effort of speakers, writers, and preachers, will be effective in securing that measure of support which the hospitals so sadly need. Mrs. Gladstone, with very great kindness and self-denial, wrote, as our readers know, an excellent appeal, which could not fail to move many hearts and consciences, and the Archbishop of Canterbury and Sir Andrew Clark's addresses at the Mansion House ought to prevent the possibility of the voluntary hospitals ever coming on the rates. But when all has been done by these and other means to nourish the growth of hospital opinion, the goal of financial success will never be reached unless the managers of the hospitals individually and collectively look the facts in the face and modify existing methods to present and future needs. It is a great thing, no doubt, to have succeeded in awakening so general an interest in hospitals as a great national system of momentous importance, but the managers must unite and co-operate with each other also, or the public interest will again cool, and a hospital rate become inevitable.

The committee and officials have a splendid field of benevolence and kindly feeling to cultivate just now; but it rests with them to improve or ruin the soil. What most laymen cannot understand is how the London hospitals and dispensaries go on living, when it is shewn that they spend every year nearly £100,000 more than they receive from their general charitable and proprietary revenues. We have looked into this question pretty closely, and we find that legacies provide the whole deficiency, on an average, in two out of every three years, so that there is at present something like £35,000 per annum needed in addition to give the London hospitals the financial strength it is the interest of all classes to secure for them. This sum would be easily obtained if the Hospital Saturday Fund Council would be wise in its methods, as we propose to show in a series of articles. They have called in the aid of eminent authorities, who have made Hospital Saturday a success in the large provincial towns; and these gentlemen, though unknown to each other, have practically advised the same course of action, as their letters show. Yet the Council does not display any sign of adapting its system to the requirements of a vast area like London, and so the hospitals are deprived of at least £30,000 per annum, which the working-classes are prepared to give if they are only properly approached as they have been in the provinces. Hospital Sunday in London might be made to produce £50,000, and Hospital Saturday at least £40,000. In this way the existing deficiency in the hospitals' exchequer might easily be wiped out for all time.

But there is, unfortunately, the further fact to be thought of, that the hospitals contain some 3,000 vacant beds every year, under the present system. If all these beds, or say five-sixths of them, were to be constantly occupied, as they might properly be throughout every day in each year, at least another £75,000 of revenue would have to be forthcoming. Can this be secured? We have no hesitation in saying that it can. How? By the simple expedient of securing the co-operation of the managers of all the London hospitals in devising a system—(*a*) to freethe out-patients' department of its present grave abuses, (*b*) to institute a certain number of paying wards, or pay beds, in several of the hospitals, and *c*) to organise a systematic canvass of the more prosperous residents in the immediate neighbourhood of each of the hospitals, with the object of securing new annual subscribers.

On the last point it may be said that, whereas in the provinces half the hospital income is derived from annual subscriptions, in London not one-tenth, and sometimes, as in the case of the great London Hospital, not one-twentieth portion of the income comes from this source. Those who attend the meeting at St. Thomas's Hospital, on Wednesday evening next at eight o'clock, will learn much about the pay system which cannot fail to interest them, and on the first point it may be said with truth that if the managers do not co-operate, and that speedily, the growth of hospital opinion to which we have referred will soon make itself felt, in a manner which may prove more forcible than pleasant. United, our great hospitals are invincible, but if they stand much longer isolated and self-contained as at present, a hospital rate is not only a possible but a probable calamity, which ought therefore, by being foreseen, to be rendered not only impossible but unnecessary.

